# The Urban Development in Relation to the Occurrence of Diseases in the Casa-Settat Region of Morocco during the Emergence of SARS-CoV-2

**DOI:** 10.1155/2022/1093956

**Published:** 2022-08-09

**Authors:** Sanaa Deblij, Bouchaib Bahlaouan, Nadia Boutaleb, FatimaZahra Boutaleb, Mohamed Bennani, Said El Antri

**Affiliations:** ^1^Hassan 2 University of Casablanca, Laboratory of Biochemistry, Environment and Agri-Food, LBEA URAC36, Casablanca 20650, Morocco; ^2^Higher Institutes of the Nursing Professions and Techniques of Health ISPITS, Casablanca 22500, Morocco; ^3^Institut Pasteur Casablanca Morocco, Laboratory of Physico-Chemical Analysis of Water, Food and Environment, Casablanca 20250, Morocco

## Abstract

The Casa-Settat region is experiencing very worrying environmental and epidemiological problems and challenges, namely, population growth, the significant development of unsupervised industrial activities, road traffic, the significant weight of the spread of diseases with high epidemiological potential such as SARS-CoV-2, the increase in hospital activities, and the significant discharge of hospital effluents highly contaminated and untreated. To understand and analyze the factors influencing the high prevalence of deaths and the occurrence of diseases under surveillance, among others SARS-CoV-2, on the quantitative data recorded relating to ten regions of Morocco, and informing, on the one hand, on intrinsic data linked to the urban development, and on the other hand, on the evolution of diseases under epidemiological surveillance, a multidimensional analysis was made. The results reveal the typological framework highlighted by the factorial map *F*1 × *F*2 which showed the individualization of the region of Casablanca explained by a large number of variables and diseases that affect it. Finally, these results call for a diagnosis that will make it possible to model new approaches and implement new actions promoting the dynamics of environmental and epidemiological change in one of the most polluted and infected regions of Morocco.

## 1. Introduction

Urban development is the reconsideration of a set of urban issues that gives a new dimension to questions relating to the quality of life for citizens and the local management of water, soil, and industry, overpopulation, transport, and accommodation. It advocates sustainable and pollution-free urban development. A region must therefore learn to properly manage and exploit its possibilities and potential while facing the various complex problems it generates [1].

**Figure 1:**
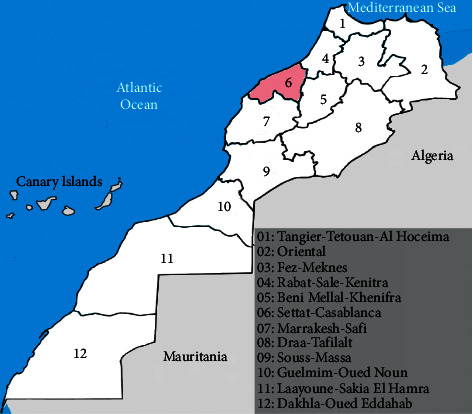
Map of Casablanca-Settat region in Morocco.

In Morocco, the Casa-Settat region (Figure 1) is the most problematic due to the challenges of uncontrolled urban development in addition to the increase in morbidity and the incidence of diseases under epidemiological surveillance, including SARS-CoV-2 [2–4].

Indeed, the Casa-Settat region declared the first patient with SARS-CoV-2 in March 2020. Since that date, it ranks first in terms of critical cases, 65% of declared cases, and high incidence rates, which have always exceeded these, recorded nationally.

**Figure 2:**
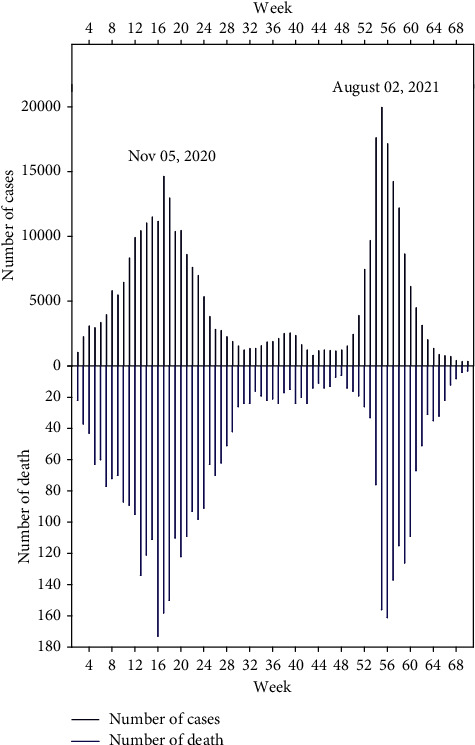
The weekly evolution of deaths and confirmed cases by SARS-CoV-2 in the Casa-Settat region from July 22, 2020, until 30/11/2021.

The start of the pandemic saw an increase of only 70 ± 10 cases per day and everything seemed in order and controllable until July, which saw a peak of 445 positive cases. Moreover, just after this date, the number of cases continued to increase to reach 2478 cases declared positive in November 02, 2020. Then, after 10 months of the pandemic and exactly between July and August 2021, the number of reported cases reached its maximum value and its large peak of 3613 cases on August 02, 2021 (Figure 2).

Also, the state of health of the Casablanca population was still declared critical and devastated with a still high number of cases of COVID-19, which represents a dangerous global pandemic officially declared on March 12, 2020, and whose number of deaths has exceeded five million of the world's population [5, 6]. The Casablanca region also experienced very high numbers of deaths which exceeded those noted in certain Moroccan regions; this number exceeded the threshold of 150 deaths during certain weeks (Figure 2).

On the other hand, and regarding population growth, the Casa-Settat region is the most populated in the Moroccan Kingdom with a total population of 7,218,021 inhabitants recorded in 2014 and concentrates the largest part of the national population with a weight of 20.3%. This situation is projected to continue until 2030 [7]. This overcrowding generates dense human activities which are the source of environmental pollution and the significant spread of diseases and epidemics and this is what is observed in the Casablanca region. Added to this, the fact that the Casablanca region represents the leading industrial and economic center in Morocco, bringing together 38.9% of the total chemical, metallurgical, glassware, agrifood, thermal energy production, textile and leather, and also plastics, and rubber industry [7].

This leading industrial and economic center is incriminated as being one of the main fixed sources of atmospheric pollution and gaseous and chemical emissions due to treatment or manufacturing processes [8].

For sulfur dioxide SO_2_ and suspended particulate matter PM10, all the air quality measurement stations located in the Casablanca region show exceedances of the SO_2_ limit value for the protection of ecosystems (20 *μ*g/m^3^) and about the health protection limit value for PM10. This testifies to great air pollution due to industrial activities and heavy road traffic, which represents 39% of vehicles registered at the national level [9]. Similarly, studies have confirmed that the increase in the number of SARS-CoV-2 cases recorded in the Casablanca region is linked to the appearance of numerous outbreaks in industrial and commercial structures [10].

Several researchers have found that pollution from industrial and road traffic due to urban development is a major cause of environmental degradation. In addition, areas close to industrial activities are marked by dangerous contamination of air, soil, and water [11–15].

The very important industrial and road traffic about urban development has a great impact on the health of the population and also the emergence of diseases such as mental disorders, cancer, and cardiovascular and lung problems [16, 17].

Typhoid fever, for example, is also more common in areas that are overcrowded and have food industry units and a faulty waste disposal system. If proper protection and control measures are taken, the occurrence of typhoid fever epidemics would be lower and well monitored [18, 19].

**Figure 3:**
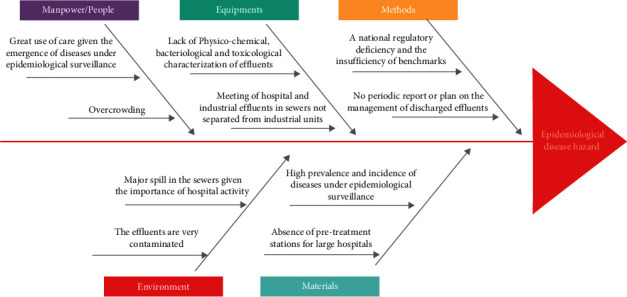
The inventory of the causes of the epidemiological disease hazard in the Casa-Settat region.

Given the burden of these diseases, the Casablanca region has the largest number of healthcare units that generate very dangerous healthcare waste. Many Moroccan studies have shown that they showed signs of significant degradation and that the majority of the physic-chemical and microbiological safety parameters studied do not comply with the Moroccan and the WHO standards [3, 20–23]. Hospital effluents in Morocco are therefore highly contaminated and can have adverse effects on human health and the ecosystem. Especially since conventional wastewater treatment technologies are not effective in eliminating these contaminants, discharged into the receiving environment [24, 25]. These liquid effluents also contain SARS-CoV-2 even in places with high ambient temperature and relatively low prevalence of COVID-19 [26]. This Ishikawa diagram identifies the inventory of the causes of the poor management of dangerous hospital effluents [27]discharged in the Casa-Settat region and it is all about uncontrolled urban development (Figure 3).

The purpose of this study is therefore to describe the impact of the various environmental problems mentioned above caused by the conditions of urban development on morbidity and the incidence of diseases and deaths caused, followed by a statistical analysis using the numerous data collected to highlight the relationship that exists between the epidemiological and environmental parameters of the region of Casa-Settat and the comparison between other Moroccan regions [28].

## 2. Materials and Methods

The data used in this analysis come mainly from the Moroccan Ministry of Health and the High Commission for Planning. The exploitation of data from the Moroccan epidemiological bulletin N°78/2021 are those from the report of the regional health department Casa-Settat from the beginning of the COVID-19 pandemic until the end of December 2021, in addition to the reproducibility of data from the monographs of the ten regions concerning the most prevalent environmental issues and the national survey on population and family health [29]. Regarding the data on the major problem of contaminated hospital effluents in the Casa-Settat region, they were extracted from a questionnaire survey with the Committees for the Fight against Nosocomial Infection within the region during a period of 4 months in 2019 [27]. All of this allowed for the detection of variables related to the epidemiological and environmental situation. The links between these highlighted variables were also studied by principal component analysis using Statistica® software to link the epidemiological situation of the regions studied to the data on urban development.

## 3. Results and Discussion

According to the data from the National Survey on Population and Family Health [30], the region of Casa-Settat is experiencing the most important health crisis (Table 1). Table 1 presents the data from the Moroccan epidemiological bulletin (2021) concerning mortality and the incidence of diseases under surveillance which targets generally health programs, especially the SARS-CoV-2 [31].

Table 2 presents the data of some urban development indicators associated with the different studied regions, data that are valid to the present day [29].

The indicators retained in the two tables attempt to define the urban characteristics of the regions in terms of the environment, health, road traffic, and industry. Once these indicators have been mastered and randomized, the focus will be on the challenges of future development without altering either the environmental component or the health component.

In this last context, the ultimate objective of the epidemiological surveillance of the diseases targeted by the Ministry of Health and which appear in Table 1 is the detection of changes in their distribution or their trend to undertake investigations and the necessary control measures and to alleviate any future social drama. Concerning cutaneous leishmaniasis as a disease under epidemiological surveillance, in Morocco, this disease is endemic and constitutes a major public health problem. According to Table 1, the Moroccan tropical and temperate zones are the most affected, namely, the Draa-Tafilalet and Marrakech-Safi regions, which have the highest number of declared cases.

These last two regions declared as active foci of leishmaniasis in Morocco are experiencing an arid climate and exceptional environmental changes and new sociodemographic conditions different from the Casablanca region (Table 2) [27]. Concerning tuberculosis is considered a major public health problem both internationally and nationally. It was declared in 2020 as the second leading cause of death, after SARS-CoV-2. Unfortunately, the Casablanca region has the highest number of declared cases at 7404 cases versus the Draa-Tafilalt region which only knows 373 declared cases during the same period (Table 1), knowing well that the Casablanca region represents the most rebellious area in Morocco, especially since this disease is found in high concentrations in neighborhoods with very high population density and periurban areas of large agglomerations [31]. For AIDS too, 64% of notified cases are found in three regions which are Souss-Massa, Casablanca-Settat, and Marrakesh-Safi. These immunocompromised patients will suffer from the high incidence and dangerousness of opportunistic infections, namely, tuberculosis, meningitis, SARS-CoV-2, and eruptive fevers which are strongly present in Casablanca region.

The cumulative number of SARS-CoV-2 deaths which is 2182 for the Casablanca region is much higher than the average recorded within the other regions and which is 731.5 and also compared to the standard deviation which is 562.25, knowing well that the number of deaths recorded in the region by SARS-CoV-2 exceeded the threshold of 150 deaths during certain weeks. Indeed, all the indicators of urban development appearing in the two tables show that there is a very significant difference between the differences between regions. Admittedly, an apparent success is reflected by Casablanca's urban attractiveness to a large extent given the concentration within the region of a large number of health structures and industrial units and road traffic followed in second place by the region Rabat-Sale-Kenitra.

Despite all this, the Casablanca region is experiencing very alarming comorbidity factors (Table 2), namely, overcrowding, air pollution, unfavorable living conditions within certain prefectures, promiscuity, the large number of healthcare structures that generate thousands of tons of healthcare waste, and thousands of liters of hospital liquid effluents, very heavy road traffic in addition to a large agglomeration of industrial and commercial units. All of these revive the risk of contamination for the people of Casablanca and make the epidemiological and environmental situation still precarious.

**Figure 4:**
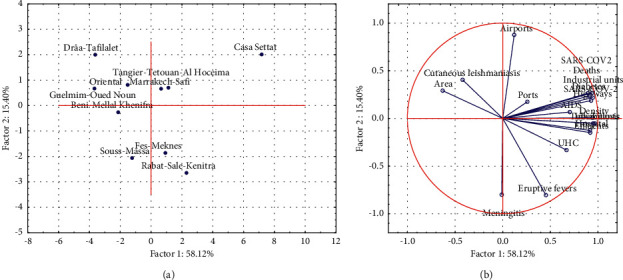
Principal component analysis. (a) The biplot and (b) correlation circle between variables and factors.

These precarious environmental consequences are therefore well identified in Table 2 and are also to be taken seriously into consideration and seriously by the decision-makers to undertake the appropriate solutions. Therefore, the tabular analysis shows a clear urban, environmental, and health divide between the Casablanca-Settat region and the rest of the other areas, which should lead to taking all the essential preventive measures to mitigate any future environmental and health crises. To synthesize and interpret all these data appearing in the tables, a statistical analysis has been developed, as shown in Figure 4.

These figures show that the regions are represented in a dispersed manner according to their epidemiological and urban development data in a two-dimensional space. This space demonstrates the impact and importance of environmental issues on the spread of certain diseases under national and regional surveillance. The region Casablanca-Settat is obviously individualized and is positioned alone on the factorial map. This is explained according to the circle of correlation with the large number of variables and diseases that affect it. This demonstrates that it is the most polluted region and the most infected by diseases and that it is under the great weight of all the epidemiological issues studied. While, the positioning of the other regions shows that they are far from the impact of pollution and the spread of diseases along an upstream-downward gradient. All these data clearly explain that the Casablanca region has a very important position and it is under the great weight of disease and pollution. The commitment to a set of actions to deal with this catastrophic situation is seen as essential to ensure a better quality of life and the well-being of citizens and future generations.

The principal component analysis (PCA) shows that the Casablanca region experiences the greatest number of diseases with high epidemiological potential. And critical environmental issues as correlated variables in Table 2 which also showed additional independent data that contributed to the second component.

In practice, it makes sense to assess the environmental challenges to study the large concentration of diseases under surveillance and at high epidemiological risk, something that the literature and the results of the PCA confirm. The Casablanca region shows a very high coefficient of variation demonstrating the impact of overcrowding, road traffic, high concentration of hospital structures, and discharged hospital effluents, on the emergence of diseases [32, 33].

Furthermore, the increase in the number of sick cases in the subject region of the study has certainly been accompanied by great hospital activity. In addition, due to the large dumping of a huge number of solid wastes and hospital liquid effluents, the circle subsequently becomes vicious. The population suffers and the environment is also in danger. Effluents have varied and dangerous characteristics, according to several studies that contain a large load of organic matter, microorganisms such as bacteria and resistant viruses, and toxic gases [34–37]. The big problem is that the industrial and health units and the overcrowding generate a great risk of infection and contamination for the population of the region and a great risk of contamination for the environment as well as a real health alert that threatens the greatest Moroccan regions as clearly demonstrated through the PCA carried out during this study.

Starting from this diagnosis and in order to concretize future preventive measures within the region, Morocco has already had an epidemiological surveillance system for several years which aims to monitor the international, national, and decentralized epidemiological situation which knows several disparities. This system must be intensified and must respond to the specificities of the region which was declared a red zone during the SARS-CoV-2 crisis by the local authorities [38]. Also, we must think about multiplying the very diversified range of preventive measures specifically for the Casablanca region, by technical procedures such as those relating to the disinfection of hotels, public places, and transport mean, up to the secure organization of the activities of industrial and commercial units, and schools, by launching mass screening campaigns in industrial units and hospitals and by monitoring contact cases [31].

The sustainability, enhancement, equipment, and staffing of the three field hospitals are built during the COVID-19 crisis in the three provinces of the Casablanca region, in this case, Benslimane, El Jadida, and Casablanca by care unit intensive, without forgetting the measures of the intensification of health control in the port and the airport of Casablanca [38]. Also, the organization of the national healthcare offers the implementation of the provisions of the health card. Continue to validate and implement regional plans for healthcare provision, taking into consideration the challenges of urbanization and environmental changes and new and reemerging health threats.

For ways to solve the problem, it would be necessary to combine sustainable development and land use planning, in this case, the creation of a green landscape and the division of space into very distinct places, devoted to housing, work, culture, and industries activities that are responsible for several social ills. Adding to this, the implementation of strategic urban planning is based on the establishment of several structuring plans for the region, namely, the urban plan, the sustainable development plan, the transport plan, and the economic development strategy by regulating any abusive activity and by introducing the “polluter pays,” where the costs of pollution control and prevention measures are borne by the polluter and those by the “user pays” [39]. Among other things, the National Strategy for Sustainable Development (SNDD) in 2030 in its eleventh strategic axis stipulates that the urban planning of overpopulated and health crisis regions, namely, the Casablanca region, should be aligned with the principles of sustainable development, which will allow generating as little pollution and waste as possible in order to meet the challenges of social and health cohesion, urban planning and environmental control, and by making the most of it [40].

## 4. Conclusion

This study is based on multidimensional statistical analysis to process numerous quantitative data from several Moroccan regions. It was therefore a question of paying more particular attention to epidemiological problems and their relationship with environmental ones in relation to the urban development data. The results show that the Casa-Settat region is the most polluted and the most infected by several dangerous diseases. In addition, it is because its urban development is not taken into consideration by political and environmental actors, this situation is becoming alarming. Several adapted prophylactic measures are to be implemented collectively and on a large scale to mitigate in the near future a great risk of the occurrence of pandemics and related deaths, as was the case for the SARS-CoV-2 pandemic that ravaged Casablanca hospitals and caused a lot of death and separation.

## Figures and Tables

**Table 1:** Distribution of diseases under epidemiological surveillance according to Moroccan regions.

Number of cases of illness	Cumulative SARS-CoV-2 deaths	SARS-CoV-2	Eruptive fevers	Tuberculosis	Meningitis	AIDS	Cutaneous leishmaniasis	Diabetes
Casa-Settat	2182	172746	8	7404	9	130	11	807
Marrakech-Safi	907	35407	5	2481	6	34	616	444
Fez-Meknes	694	19100	20	3499	41	11	189	383
Tangier-Tetouan-Al Hoceima	739	38404	9	4433	2	2	39	296
Souss-Massa	500	31677	12	1834	107	72	118	41
Beni-Mellal-Khenifra	448	15847	5	1215	18	35	359	151
Oriental	666	28016	7	1391	24	16	129	274
Draa-Tafilalet	312	14529	2	373	26	3	2098	264
Guelmim-Oued Noun	104	6182	0	1834	5	11	0	34
Rabat-Sale-Kenitra	763	66467	25	5186	87	35	75	436

**Table 2:** Distribution of Moroccan regions according to their related epidemiological issues: constructed from data from monographs of 10 Moroccan regions.

Regions	Inhabitants	Density	Hospital structures	Highways	Industrial units	Area	Daily effluents (liters per day)	Ports	Airports	CHU
Casa-Settat	**7 218 021**	**371**	**24**	**945.5**	**3 097**	**19 448**	**12700**	**3**	3	1
Marrakech-Safi	4 687 947	119	15	453	674	39 167	8200	2	2	1
Fes-Meknes	4 347 958	108	21	202	1014	40 075	11200	0	1	1
Tangier-Tetouan-Al Hoceima	3 725 192	215.8	16	226	878	17 262	8700	15	3	1
Souss-Massa	2 817 204	52	8	250	396	53 789	4700	1	1	1
Beni-Mellal-Khenifra	2 581 703	90	9	0	315	28 374	4500	0	1	0
Oriental	2 402 374	27	10	171	401	88 681	5700	3	3	1
Guelmim-Oued Noun	441 800	9.5	4	0	24	46 108	2000	2	2	0
Draa-Tafilalt	1 673 773	18.8	8	0	62	88 836	4 000	0	3	0
Rabat-Sale-Kenitra	4 769 423	262	19	304.5	776	18 194	10 200	3	1	1

## Data Availability

The data used to support the findings of this study are available from the corresponding author upon request.
